# Inhibitory GABAergic Neuron Loss due to Oxidative Damage During Ex Vivo Acute Brain Slice Preparation Influences Genesis and Dynamics of Epileptiform Activity

**DOI:** 10.1111/jnc.70367

**Published:** 2026-01-28

**Authors:** Felix Chan, Anupam Hazra, Ashan Jayasekera, Katherine Huang, Shuna Whyte, Leolie Telford‐Cooke, Kamilah Lakhani, Xiaomeng Li, Rebecca Shields, Angeline Kosim, Darwin Su, Carol Murray, Mark O. Cunningham

**Affiliations:** ^1^ Institute of Neuroscience, Medical School, Faculty of Medical Sciences Newcastle University Newcastle upon Tyne UK; ^2^ Department of Pharmacy, School of Health Sciences, College of Medicine and Health University of Birmingham Birmingham UK; ^3^ Birmingham Centre for Neurogenetics University of Birmingham Birmingham UK; ^4^ Birmingham Centre for Human Brain Health University of Birmingham Birmingham UK; ^5^ Discipline of Physiology, School of Medicine Trinity College Dublin Dublin Ireland; ^6^ FutureNeuro Research Ireland Centre for Translational Brain Science RCSI University of Medicine & Health Sciences Dublin Ireland

**Keywords:** brain slice, dynamics, Electrophysiology, epilepsy, inhibitory neuron, oxidative stress

## Abstract

Ex vivo acute brain slice is a popular technique in neuroscience research with many variations. While many variations are currently used by labs around the world, no study has comprehensively examined the impact of these variations on the quality of the acute brain slice preparation. In this study, we compared different animal sacrifice methods (decapitation or transcardial perfusion) and cutting solution (normal or sucrose artificial cerebrospinal fluid). Brain slices were prepared from 10 to 12 weeks old male Wistar rats ( *Rattus norvegicus* ). Neuronal population was quantified by immunohistochemistry against various neuronal markers. Neuronal dynamics was evaluated by in vitro electrophysiology using two acute epilepsy models—zero‐magnesium and 4‐aminopyridine. We found that the method of brain slice preparation significantly affected the quality of the brain slice preparation. In general, the combination of transcardial perfusion and sucrose artificial cerebrospinal fluid produces the optimal brain slice preparation. The slices prepared with transcardial perfusion and sucrose aCSF had higher preservation of inhibitory interneurons and subsequently less successful induction of acute epileptiform activity. We also found that loss of inhibitory GABAergic neurons during brain slice preparation is primarily due to oxidative damage. Limiting oxidative stress is an effective neuroprotection strategy to prevent loss of inhibition in brain slice preparation. In conclusion, consideration of brain slice preparation method is crucial in preserving inhibitory GABAergic neurons and the degree of inhibition in the slice. Loss of inhibitory interneuron due to oxidative stress significantly affects quality of brain slice preparation and subsequent ex vivo epileptiform activity induction and dynamics.

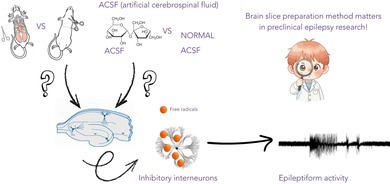

## Graphical abstract

Ex vivo brain slice preparation is a popular technique in neuroscience research with many variations in preparation method. We compared two popular variations in brain slice preparation and found that the choice of brain slice preparation technique significantly affects the viability of neuronal preparation. We found that inhibitory interneurons are particularly affected during brain slice preparation due to oxidative stress damage. The loss of inhibitory interneurons during brain slice preparation significantly affected the brain slice network and the epileptiform activity it generates in the acute epilepsy induction model. Neurochemistry research should account for the impact of brain slice preparation on neuronal physiology.

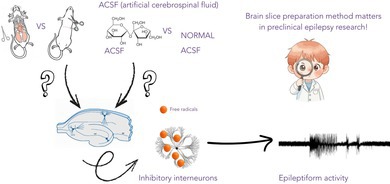

**Abbreviations**
4‐AP: 4‐aminopyridine
ACSF: artificial cerebrospinal fluid
DA: decapitation with normal artificial cerebrospinal fluid with antioxidants
DC: decapitation with normal artificial cerebrospinal fluid with chrysin
DN: decapitation with normal artificial cerebrospinal fluid
DNPH: 2‐dinitrophenylhydrazine
DS: decapitation with sucrose artificial cerebrospinal fluid
GABA: gamma‐aminobutyric acid
HIF: hypoxia‐inducible factor
LRD: late‐recurrent discharge
LTP: long term potentiation
nACSF: normal artificial cerebrospinal fluid
NMDA‐R: N‐methyl‐D‐aspartate receptor
PN: perfused with normal artificial cerebrospinal fluid
PS: perfused with sucrose artificial cerebrospinal fluid
RRID: Research Resource Identifier
sACSF: sucrose artificial cerebrospinal fluid
SLE: seizure‐like event

## 1 Introduction

Since its inception in 1957 by Li and McIlwain (Li and McIlwain 1957), ex vivo acute brain slice electrophysiology has proven to be a very popular technique in interrogating the functional state of brain activity. Many modifications to this technique have existed since then—one of import is the use of sucrose artificial cerebrospinal fluid (sACSF) to reduce excitotoxicity in brain slices (Aghajanian and Rasmussen 1989). Slices prepared in sACSF have also reported differences in the functional state of brain slices, including a significant change in long‐term potentiation (LTP) induction and gamma‐aminobutyric acid (GABA)‐ergic neurotransmission (Avegno et al. 2019; Kuenzi et al. 2000). Collectively, it can be said that the composition of the ACSF as the cutting solution in brain slice preparation directly influences neuronal survival and function; and ultimately the quality of the brain slice preparation (Richerson and Messer 1995).

Another major adaptation in the slice preparation technique is the method of animal sacrifice and brain extraction. Classically, animals such as rodents were simply decapitated with or without anesthetic agents, and brain was obtained speedily from this preparation. Indeed, this method is still used presently and can still be seen in published standard operating protocol for brain slice preparation (Papouin and Haydon 2018). Another popular method of euthanasia is transcardial perfusion of rodents. Typically used to perfuse fixative to preserve brain tissue for histology (Wu et al. 2021), transcardial perfusion of ACSF, either nACSF or a modified ACSF such as sACSF, has been used to prepare acute brain slices for electrophysiology purposes (Avegno et al. 2019; Chan et al. 2019). Many other variations to this technique also exist, such as the anesthetic agent used (Schurr et al. 1995), temperature of slicing, and slicing techniques; and while an attempt has been made at creating a consensus and standard of practice for ex vivo brain slice preparation (Raimondo et al. 2017), individual research groups still maintain their own standard operating procedure for brain slice electrophysiology.

In the modern age of neuroscience, the brain slice preparation is a highly versatile tool that can be used to study brain activity and function. In addition to its use in acute preparation, brain slices can be organotypically cultured to extend the viability of the brain slices beyond the day of preparation. Organotypic brain slices have enabled the study of chronic network remodeling (Albus et al. 2013) and the use of genetic toolkits to dissect specific cellular contributions to network activity (Toth et al. 2021). In addition to its established use for ex vivo electrophysiology, brain slice preparations have also been used for complementary techniques using advanced optical tools, such as calcium imaging (Parrish et al. 2018), voltage‐sensitive dye imaging (Carlson and Coulter 2008), and optogenetics (Kashima et al. 2024). Thus, understanding the optimal brain slice preparation technique is an important and timely question for the field.

Epilepsy is a major area of neuroscience research where ex vivo acute brain slice electrophysiology is commonly used. A recent report from the International League Against Epilepsy Task Force clearly recognized the importance of using acute brain slice preparation as a model for epilepsy study, particularly for high throughput screening and therapeutic development (Morris et al. 2023). However, as mentioned previously, the use of acute brain slice model of epilepsy varies by method of induction, variations in the protocol, and experimentation in specific research groups (Raimondo et al. 2017). The different acute models of epilepsy target different methods of induction; such as increasing N‐methyl‐D‐aspartate receptor (NMDA‐R) mediated excitation using the zero magnesium (0 Mg^2+^) model (Mody et al. 1987), increasing potassium‐channel activity using 4‐aminopyridine (Brückner and Heinemann 2000), or reducing GABAergic inhibition using GABA_A_ receptor antagonists (for e.g., picrotoxin, pentylenetetrazole or bicuculline (Hashimoto et al. 2017; Müller et al. 2018; Samoilova et al. 2003)). Despite the well‐established model of epilepsy in acute brain slice preparation, no study has directly evaluated the influence of different methods of acute brain slice preparation on the induction and dynamics of epileptiform activity. This is particularly surprising given that, as mentioned in the aforementioned studies, change in the method of brain slice preparation can have a dramatic effect on synaptic properties, inhibition, and neuronal survival—all of which can affect the epileptiform activities induced.

This study fills in this important gap in our knowledge by comprehensively examining the impact of different brain slice preparation techniques on the dynamics and induction of epileptiform activity. We will focus on two aspects of brain slice preparation techniques which we hypothesize pose the most significant determinant factor: (1) the composition of the cutting solution; either nACSF or sACSF and (2) transcardial perfusion versus decapitation. Of interest to us is specifically the effect of this variation in brain slice preparation techniques on the inhibitory interneuron population in the brain given their importance in shaping the epileptic brain network.

## 2 Methods

### 2.1 Ethical Approval and Use of Animals

Adult (10–12 weeks old) male Wistar rats (RRID:RGD_23123472) weighing circa 300‐320 g were used in this study. All animal handling and experimentation were done in accordance with the requirements of the United Kingdom Animals (Scientific Procedures) Act 1986. All animals were purchased from Charles River Laboratories UK. Animals were housed in a A RM1 cage; housed in groups of 4 on a 12 h light–dark cycle under controlled conditions (temperature: 20°C–25°C; humidity: 40%–60%). Food and water were available ad libitum. A total of 72 animals were used in this study. Separate cohorts were used for the immunostaining (12 animals) and electrophysiology experiments (60 animals). Breakdown of animals per group is provided in the figure legend for each experiment. Animals were arbitrarily selected for each experimental group.

### 2.2 Brain Slice Preparation

Brain slices used for the in vitro studies were prepared using either one of the following protocols. In the first paradigm, which we termed “decapitated,” the animals were exposed to inhaled isoflurane (IsoFlo, Abbott Laboratories Ltd. cat. no. C2485) and after deep anesthesia was achieved, cervical dislocation was performed. The rats were then decapitated and brains were rapidly removed in cutting solution. In the second paradigm, which we termed ‘perfused’, the animals were again exposed to inhaled isoflurane and terminal anesthesia was given by injection of 30 mg of ketamine (Narketan, Vetoquinol cat. no. 08007/4090) and 6 mg of xylazine (Xylacare, Animalcare cat. no. 32742/4036). After all reflexes were lost, we performed transcardial perfusion of the cutting solution. The brain was then rapidly removed, again, in the cutting solution.

Two cutting solutions were tested in this study, namely sACSF, which contains 252 mM sucrose (Fisher Scientific cat. no. 10346150), 24 mM NaHCO_3_ (Merck Sigma cat. no. S6014), 2 mM MgSO_4_ (Merck Sigma cat. no. M7506), 2 mM CaCl_2_ (Merck Sigma cat. no. C5670), 3.5 mM KCl (Merck Sigma cat. no. P3911), 1.25 mM NaH_2_PO_4_ (Merck Sigma cat. no. S0751), and 10 mM glucose (Avantor cat. no. 47249); and nACSF, which contains 126 mM NaCl (Merck Sigma cat. no. S9888), 24 mM NaHCO_3_, 1.2 mM MgSO_4_, 1.2 mM CaCl_2_, 3 mM KCl, 1.25 mM NaH_2_PO_4_, and 10 mM glucose. All the cutting solutions were prepared ice‐cold and oxygenated (95% O_2_/ 5% CO_2_).

By combining two slice preparation methods with two different cutting solutions, we systematically tested four variations of brain slice preparation techniques, namely (1) decapitated with sACSF (DS), (2) decapitated with nACSF (DN), (3) perfused with sACSF (PS), and (4) perfused with nACSF (PN). The remaining slice preparation protocol is the same for any of the four paradigms. The brains were sliced using a vibratome (5100 mz, Camden Instruments) in the transverse orientation (450 μm thickness). Slices containing the entorhinal cortex were collected and placed in an interface holding chamber in oxygenated nACSF at room temperature for 1 h before any experimentation.

For the rescue studies, two additional cutting solutions were utilized: (1) a nACSF solution containing a cocktail of antioxidants (10 mM ascorbic acid (Merck Sigma cat. no. 1043003), 100 μM alpha‐tocopherol (Merck Sigma cat. no. 258024), and 2 mM N‐acetylcysteine (Merck Sigma cat. no. A7250)) (resulting in a paradigm called DA—when combined with decapitation) and (2) a nACSF solution containing chrysin (Merck Sigma cat. no. C80105) (0.05% v/v)—called chrysin cutting solution (resulting in a paradigm called DC—when combined with decapitation).

### 2.3 Electrophysiology

For local field potential recordings, brain slices were transferred to interface recording chamber maintained at 30°C–33°C which is continuously perfused with oxygenated nACSF solution. Slices were left to incubate in the recording chamber for 30 min. To evoke epileptiform activity, we used two commonly studied in vitro models of seizures: (1) zero Mg^2+^ model (where a Mg^2+^‐free aCSF formulation is used) and 4‐aminopyridine (Merck Sigma cat. no. A78403) (4‐AP; 200 μM) model. Local field potentials were recorded with aCSF filled microelectrodes positioned in the layer 2–3 of the medial entorhinal cortex (mEC). The signal was amplified using the EXT10‐2F differential amplifier (NPI Electronic), filtered online (0.1‐500 Hz), passed through a 50 Hz noise eliminator (Hum‐Bug, Quest Scientific), digitized by ITC‐18 acquisition board (Instrutech), sampled at 5 kHz, and acquired using Axograph X (Axograph Scientific) software. All electrophysiological recordings were analyzed using AxoGraph X software by an experimenter blinded to the experimental conditions.

### 2.4 Immunohistochemistry

For immunohistochemistry, brain slices were prepared according to the different slice preparation paradigm as described above and incubated in the holding chamber for 3 h. Following this, the slices were fixed in 4% paraformaldehyde in 0.1 M phosphate‐buffered saline (Santa Cruz cat. no. sc‐281 692) and stored at 4°C until processing. Brain slice processing and immunohistochemistry was conducted using a previously published protocol (Chan et al. 2019). The primary and secondary antibodies used in this study were listed in Tables S1 and S2. Sections were visualized using a stereology light microscope (Olympus, BX51), and the software StereoInvestigator (MBF Bioscience) was used to perform the cell count. A region was drawn on the medial entorhinal cortex layers I, II, and III of each brain section, and counts were normalized by the area of the region to give cell density (cells/mm^2^) values. Count was performed by an independent investigator blinded to the treatment group.

### 2.5 Biochemical Assay

For the biochemical assay, brain slices were prepared according to the different slice preparation paradigm as described above and incubated in the holding chamber for 3 h. Four brain slices were homogenized into one pooled sample using the T‐18 digital Ultra‐Turrax blender (IKA Labortechnik) through a 3 × 15 s blend cycle, with 30 s break in‐between, in 1 mL of ice‐cold MES buffer (50 mM MES, 1 mM EDTA, 1% Tween‐20, 1 mM PMSF, and 1× protease inhibitor cocktail). The resulting homogenate was then centrifuged at 10000×*g* for 10 min at 4°C to separate the insoluble fraction. The supernatant was extracted and snap‐frozen at −80°C until processed. Carbonyl detection was performed as per established method on derivatization with 2,4‐dinitrophenylhydrazine (DNPH) and detected through colorimetric assay (Levine et al. 1990). The absorbance was read at 370 nm using the SpectraMax M3 plate reader (Molecular Devices) and was corrected by subtracting the control absorbance from the sample absorbance for each sample. The corrected protein carbonyl value was then normalized against the protein concentration as measured by a standard Bradford assay.

### 2.6 Statistical Analysis

Data are displayed as mean ± SEM. Normality of the dataset was tested using the Shapiro–Wilk test. Statistical significance testing for normally distributed datasets was conducted using either a two‐tailed *t*‐test or ANOVA (one‐factor or two‐factor) with post hoc *t*‐test and correcting for multiple comparisons. In cases of datasets that were not normally distributed, equivalent non‐parametric tests were utilized. Statistical significance was accepted at *p* < 0.05. No test for outliers was conducted and no data points were excluded. All statistical analysis was conducted in GraphPad Prism version 10. No sample size was calculated a priori. Sample size was determined based on previous brain slice electrophysiology of a similar nature from our laboratory (Chan et al. 2019). In this study, with an effect size of 0.4, a *n*‐value of 6 slices achieved a 97% power in two‐tailed *t*‐test comparison, justifying the sample sizes used in this study.

## 3 Results

### 3.1 Neuronal Viability Varies With Brain Slice Preparation Method

**FIGURE 1:**
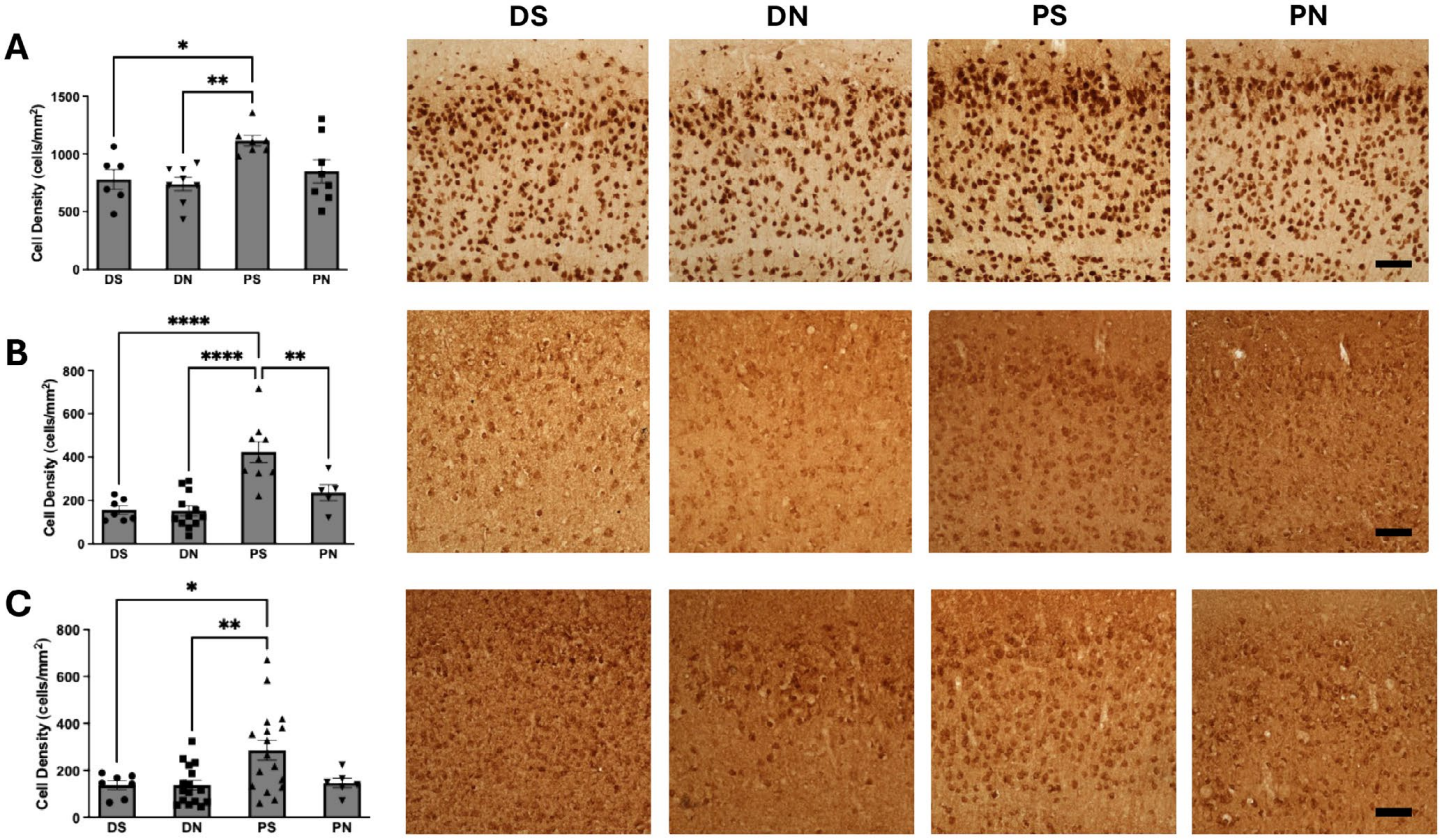
Differing slice preparation methods alter neuronal population in vitro. In each panel is shown representative photomicrographs of the staining profile for NeuN (A), GABA (B), and CaMKII (C) respectively. Alongside the photomicrographs, a bar chart quantifying the cell density of each slice preparation group is shown with statistics. DS—decapitated with sucrose aCSF, DN—decapitated with normal aCSF, PS—perfused with sucrose aCSF, PN—perfused with normal aCSF. *p < 0.05, **p < 0.01, ***p < 0.001, **** *p* < 0.0001. One‐way ANOVA was used. Number of slices (*n*) for DS = 6–7 slices from 4 rats, DN = 8–16 slices from 4 rats, PS = 7–17 slices from 4 rats, PN = 5–8 slices from 4 rats. Error bars represent SEM. Scale bar—10 μm.

We comprehensively assessed the viability of neurons in different methods of brain slice preparations (see Figure 1) firstly by detecting the expression of constitutively expressed nuclear protein, NeuN. Brain slices prepared using perfusion with sucrose aCSF (PS) (1115.0 ± 46.5 cells/mm^2^) had the largest number of NeuN‐positive cells and this was significantly greater (one‐way ANOVA, *F*(3,25) = 4.70, *p* = 0.0091) than brain slices prepared using decapitation with normal ACSF (DN) (738.5 ± 58.1 cells/mm^2^) or decapitation with sucrose ACSF (DS) (850.1 ± 99.9 cells/mm^2^). This significant reduction in NeuN expression indicated that there was a significant loss of neurons in the brain slices prepared using the decapitation method. As NeuN is expressed in both glutamatergic and GABAergic neurons, we next examined, using antibodies to Ca^2+^/calmodulin‐dependent protein kinase II (CaMKII) and GABA, if the observed reduction in neuronal population was specific to excitatory or inhibitory neurons respectively. Brain slices prepared using the PS method (423.5 ± 48.4 cells/mm^2^) exhibited the largest immunoreactivity for GABA and this was significantly greater (one‐way ANOVA, *F*(3,29) = 15.10, *p* < 0.0001) than any of the other methods (DN; 152.3 ± 23.7 cells/mm^2^, DS; 155.8 ± 18.84 cells/mm^2^, PN; 236.2 ± 36.8 cells/mm^2^). Similarly, the largest immunoreactivity for CaMKII was observed in brain slices prepared using the PS method (285.6 ± 42.7 cells/mm^2^) and this was significantly greater (one‐way ANOVA, *F*(3,42) = 5.15, *p* = 0.0040) than slices prepared using DN (137.1 ± 20.9 cells/mm^2^) or DS (137.1 ± 18.7 cells/mm^2^) method.

### 3.2 Specific Inhibitory Interneuron Subtypes Are Selectively Lost Across Different Brain Slice Preparation Methods

There is a high degree of heterogeneity in terms of the physiology, anatomy, and molecular phenotype of cortical interneurons (Lim et al. 2018). Results in the previous section demonstrated a profound difference in the number of inhibitory GABAergic interneurons in acute brain slices depending on the slice preparation methods. We next aimed to examine if a specific subtype of interneuron is preferentially affected by the difference in the method of brain slice preparation. This is done by examining the immunoreactivity for various calcium binding proteins that serve as biomarkers for discrete populations of inhibitory interneurons.

**FIGURE 2:**
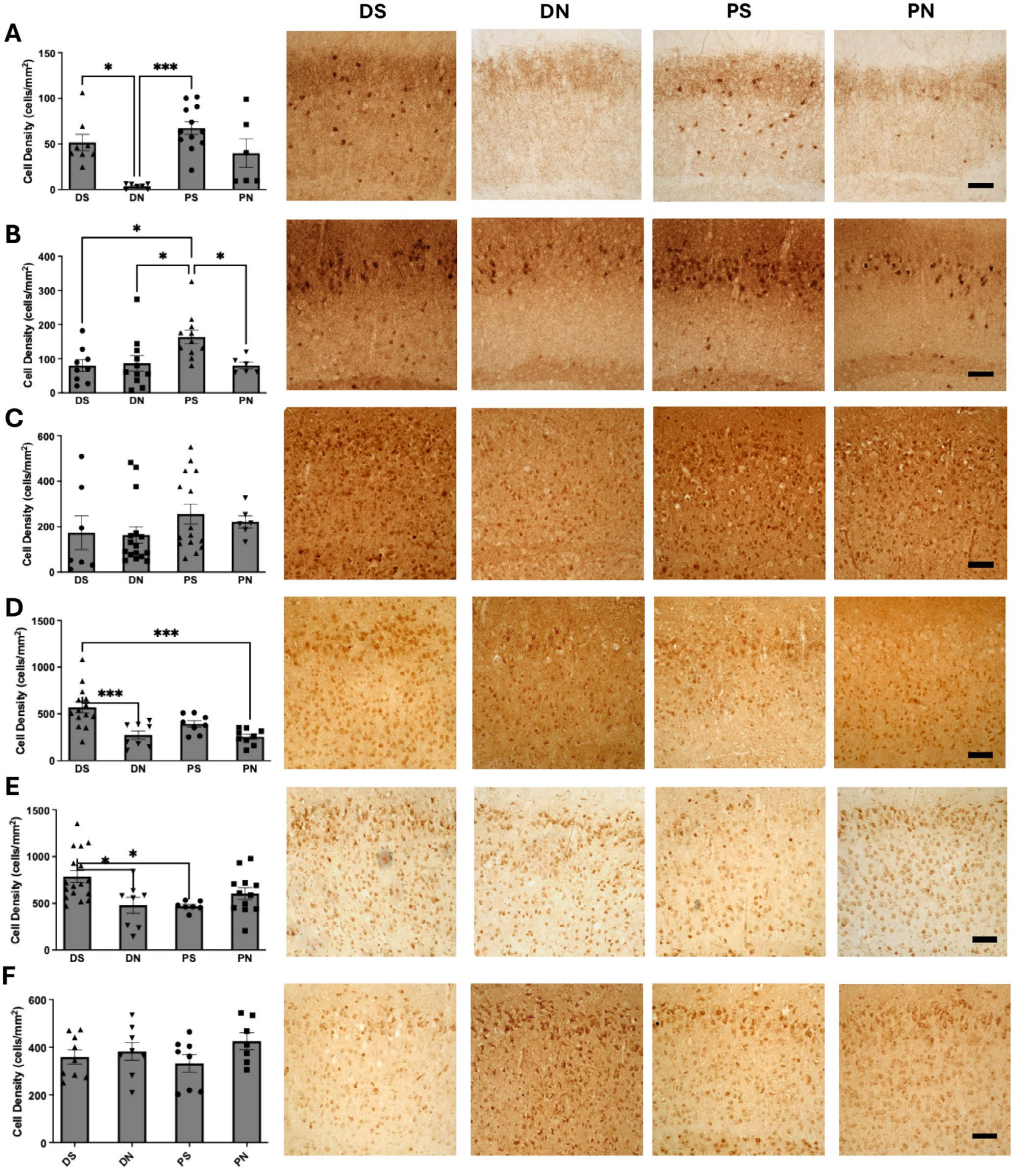
Inhibitory interneuron subtypes are differentially affected by variations in slice preparation methods. In each panel is shown representative photomicrographs of the staining profile for parvalbumin (A), calbindin (B), calretinin (C), VIP (D), CCK (E), and somatostatin (F) respectively. Alongside the photomicrographs, a bar chart quantifying the cell density of each slice preparation group is shown with statistics. DS, decapitated with sucrose aCSF; DN, decapitated with normal aCSF; PS, perfused with sucrose aCSF; PN, perfused with normal aCSF. *p < 0.05, **p < 0.01, *** *p* < 0.001. *n* for DS—8, DN—7, PS—12, PN—6. Kruskal–Wallis was used for A and C and One way ANOVA was used for B and D–F. Number of slices (*n*) for DS = 6–8 slices from 4 rats, DN = 6–16 slices from 4 rats, PS = 7–12 slices from 4 rats, PN = 6–12 slices from 4 rats. Error bars represent SEM. Scale bar—10 μm.

Interestingly, we found that parvalbumin‐expressing (PV^+^) interneurons are significantly affected (Kruskal–Wallis, *H* = 18.75, DF = 3, *p* = 0.0003) by slice preparation methods, with brain slices prepared using the DN method demonstrating the lowest density of PV^+^ interneurons (DN; 3.7 ± 1.0 cells/mm^2^) which was significantly lower than slices prepared using the DS or PS method (DS; 51.6 ± 9.0 cells/mm^2^, PS; 67.6 ± 7.0 cells/mm^2^). Slices prepared using the PS method showed the highest density of calbindin‐expressing interneurons (PS; 164.0 ± 20.3 cells/mm^2^) which was significantly higher (one‐way ANOVA, *F*(3,33) = 4.45, *p* = 0.0099) than slices prepared with any other preparation methods (DN; 86.7 ± 22.6 cells/mm^2^, DS; 80.3 ± 17.2 cells/mm^2^, PN; 80.5 ± 9.9 cells/mm^2^). Slices prepared using the DS method showed enrichment of both vasoactive intestinal peptide (VIP^+^)‐ and cholecystokinin (CCK^+^)‐expressing interneurons. Specifically, the VIP^+^ interneurons in DS‐prepared slices (DS; 392.6 ± 35.8 cells/mm^2^) were significantly higher (one‐way ANOVA, *F*(3,35) = 9.49, *p* < 0.0001) than DN‐ and PN‐prepared slices (DN; 273.9 ± 45.4 cells/mm^2^, PN; 255.7 ± 27.8 cells/mm^2^). The CCK^+^ interneurons in DS‐prepared slices (DS; 787.3 ± 63.6 cells/mm^2^) were significantly higher (one‐way ANOVA, *F*(3,40) = 5.14, *p* = 0.0042) than DN‐ and PS‐prepared slices (DN; 479.5 ± 84.6 cells/mm^2^, PS; 469.9 ± 19.9 cells/mm^2^). There was no significant difference in the expression of calretinin (CR^+^)‐expressing (Kruskal–Wallis, *H* = 5.90, DF = 3, *p* = 0.1168) or somatostatin (SOM^+^)‐expressing interneurons (one‐way ANOVA, *F*(3,28) = 1.20, *p* = 0.3276) among the different brain slice preparation methods (Figure 2).

### 3.3 Interneuron Preservation Across Slice Preparation Methods Affects Network Excitability and Function

Given the significant differences in the level of inhibitory and excitatory neuron composition following different brain slice preparation methods, we hypothesized that this would result in a difference in the network excitability and function. We tested this using an acute epileptiform induction model—the 0 Mg^2+^ model, a widely established model to study seizure initiation and dynamics in vitro (Anderson et al. 1986). The 0 Mg^2+^ model induces epileptiform activity by increasing excitability via excessive activation of the NMDAR following removal of the voltage‐dependent block mediated by the Mg^2+^ ions (Fekete and Wang 2025). As different brain slice preparation methods influence excitatory‐inhibitory balance, we speculated that the probability of seizure induction and its dynamics would be affected by the variations in this methodology.

**FIGURE 3:**
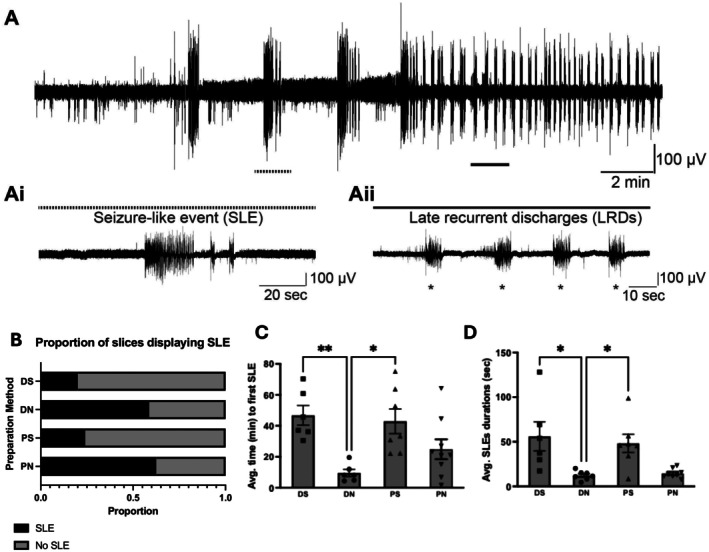
Seizure‐like event (SLE) induction is significantly affected by variation in brain slice preparation method in the 0 Mg^2+^ model. In panel A is shown an example trace showing typical early induction of SLEs—zoomed in at Ai—and transitioning to late recurrent discharges (LRDs)—zoomed in at Aii. Shown in B is the proportion of slices displaying SLE arranged by different brain slice preparation methods. Shown in C and D are the average time to first SLE (C) in minutes and average SLE durations (D) in seconds. DS, decapitated with sucrose aCSF; DN, decapitated with normal aCSF; PS, perfused with sucrose aCSF; PN, perfused with normal aCSF. *p < 0.05, ** *p* < 0.01, *n*'s for DS: 6, DN: 7, PS: 7, PN: 9. Chi‐squared test was used for B and Kruskal–Wallis test for C and D. Number of slices (*n*) for DS = 6 slices from 4 rats, DN = 6 slices from 7 rats, PS = 7 slices from 4 rats, PN = 9 slices from 4 rats. Error bars represent SEM.

The 0 Mg^2+^ model induces two forms of activity—in the early phase, seizure‐like events (SLEs) are recorded (Jones 1989; Jones et al. 1992); and after some regular occurrences of these SLEs, the network transitioned towards late recurrent discharges (LRDs) (Li Zhang et al. 1995)—see Figure 3A. Interestingly, variations in the preparation methodology produced a significant difference in the proportion of slices displaying SLE, with slices prepared using the DN method more likely to experience SLE in the 0 Mg^2+^ model, *x* ^2^ (3, *N* = 70) = 18.2; *p* = 0.0004. However, although the SLE induction probability in DS, PS, or PN are similar, the dynamics of the SLEs induced vary. The time to induce SLEs is significantly lower (Kruskal–Wallis, *H* = 14.79, DF = 3, *p* = 0.0020) in slices prepared using the DN method (DN; 9.5 ± 2.3 min) compared against DS or PS methods (DS; 46.8 ± 6.4 min, PS; 42.9 ± 8.0 min). The duration of SLEs is also significantly lower (Kruskal–Wallis, *H* = 14.81, DF = 3, *p* = 0.0020) in slices prepared using the DN method (DN; 12.2 ± 1.9 s) compared against DS or PS methods (DS; 56.0 ± 16.2 s, PS; 48.2 ± 10.3 s). SLE dynamics did not differ significantly between the DN and PN methods, suggesting that the cutting solution composition has a greater impact on SLE induction.

To ensure that the observed change in network activity is not specific to the 0 Mg^2+^ model, we also tested the use of 4‐AP acute epileptiform induction model in the various brain slice preparation methods. 4‐AP blocks voltage‐gated potassium channels, which reliably induce seizure‐like events (SLEs) in acute brain slice preparation (Barbarosie and Avoli 1997; Barbarosie et al. 2002; Heuzeroth et al. 2019). 4‐AP induces SLEs similar to those in the zero‐magnesium model, but via distinct induction mechanisms.

**FIGURE 4:**
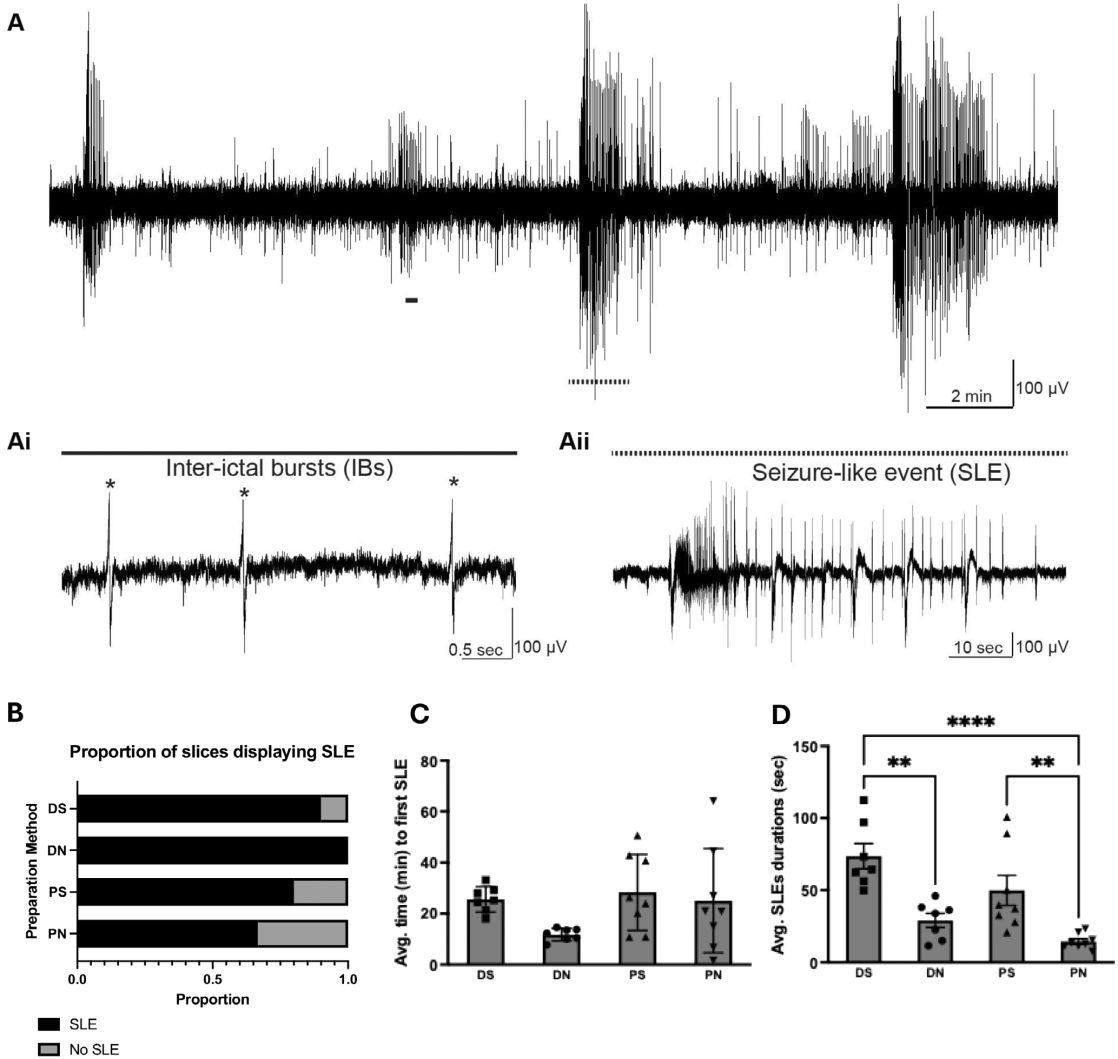
Seizure‐like event (SLE) induction is significantly affected by variation in brain slice preparation method in the 4‐AP model. In panel A is shown an example trace showing typical traces from 4‐AP induction with differences in epileptiform activity shown in PS and DN. Shown in B is the proportion of slices displaying SLE arranged by different brain slice preparation methods. Shown in C and D are the average time to first SLE (C) in minutes and average SLE durations (D) in seconds. DS, decapitated with sucrose aCSF; DN, decapitated with normal aCSF; PS, perfused with sucrose aCSF; PN, perfused with normal aCSF. **p < 0.01, ****p < 0.0001, *n* for DS—7, DN—7, PS—8, PN—8. Chi‐squared test was used for B and ANOVA test for C and D. Number of slices (*n*) for DS = 7 slices from 7 rats, DN = 7 slices from 7 rats, PS = 8 slices from 8 rats, PN = 7 slices from 4 rats. Error bars represent SEM.

The 4‐AP model induces two forms of activity: inter‐ictal bursts and seizure‐like event (SLE) similar in nature to the SLE in the 0 Mg^2+^ model—see Figure 4A. Given the similarity of the SLE in the 4‐AP model to the SLE in the 0 Mg^2+^ model, we chose to analyze and quantify the parameters of the SLEs induced by 4‐AP in the different brain slice preparation methods. Indeed, variation in brain slice preparation method similarly causes a significant difference in the proportion of slices displaying SLE, with slices prepared using DN method always consistently experiencing SLE on the 4‐AP model—unlike all the other slice preparation methods, *x* ^2^ (3, *N* = 94) = 34.1; *p* < 0.0001. Interestingly, the time to induce SLEs is not significantly different (one‐way ANOVA, *F*(3,26) = 2.21, *p* = 0.1109) between the different brain slice preparation methods, although slices prepared using DN methods showed a trend towards an earlier SLE induction time, as in the 0 Mg^2+^ model. The duration of SLEs is significantly affected (one‐way ANOVA, *F*(3,26) = 12.29, *p* < 0.0001), with slices prepared using DN method (DN; 29.1 ± 4.9 s) and PN method (PN; 14.5 ± 1.8 s) exhibiting shorter duration of SLEs compared against their sACSF prepared counterparts (DS; 73.7 ± 8.7 s, PS; 49.9 ± 10.4 s). Regardless of the epilepsy induction model, the method of brain slice preparation significantly influenced both the likelihood of successful SLE induction and the dynamics of the resulting events.

### 3.4 Limiting Oxidative Damage During Brain Slice Preparation Can Preserve Inhibitory Interneurons

Due to their intrinsically high firing rates (in some cases > 100 Hz), inhibitory interneurons impose significant metabolic demands on the brain (Kageyama and Wong‐Riley 1982; Wang and Michaelis 2010). This leads to inhibitory interneurons being particularly vulnerable to oxidative damage (Wang and Michaelis 2010), a natural byproduct of high levels of oxidative metabolism. To rescue the loss of inhibitory interneurons, we supplemented the DN cutting solution with potent antioxidant combinations, namely ascorbic acid (Gęgotek and Skrzydlewska 2022), α‐tocopherol (Robeson and Baxter 1943), and N‐acetylcysteine (Ezeriņa et al. 2018). These antioxidant combinations were chosen as they have been successfully incorporated into cutting solution compositions that were formulated specifically for preserving ex vivo human brain slices (Jones et al. 2016; Ting et al. 2018).

**FIGURE 5:**
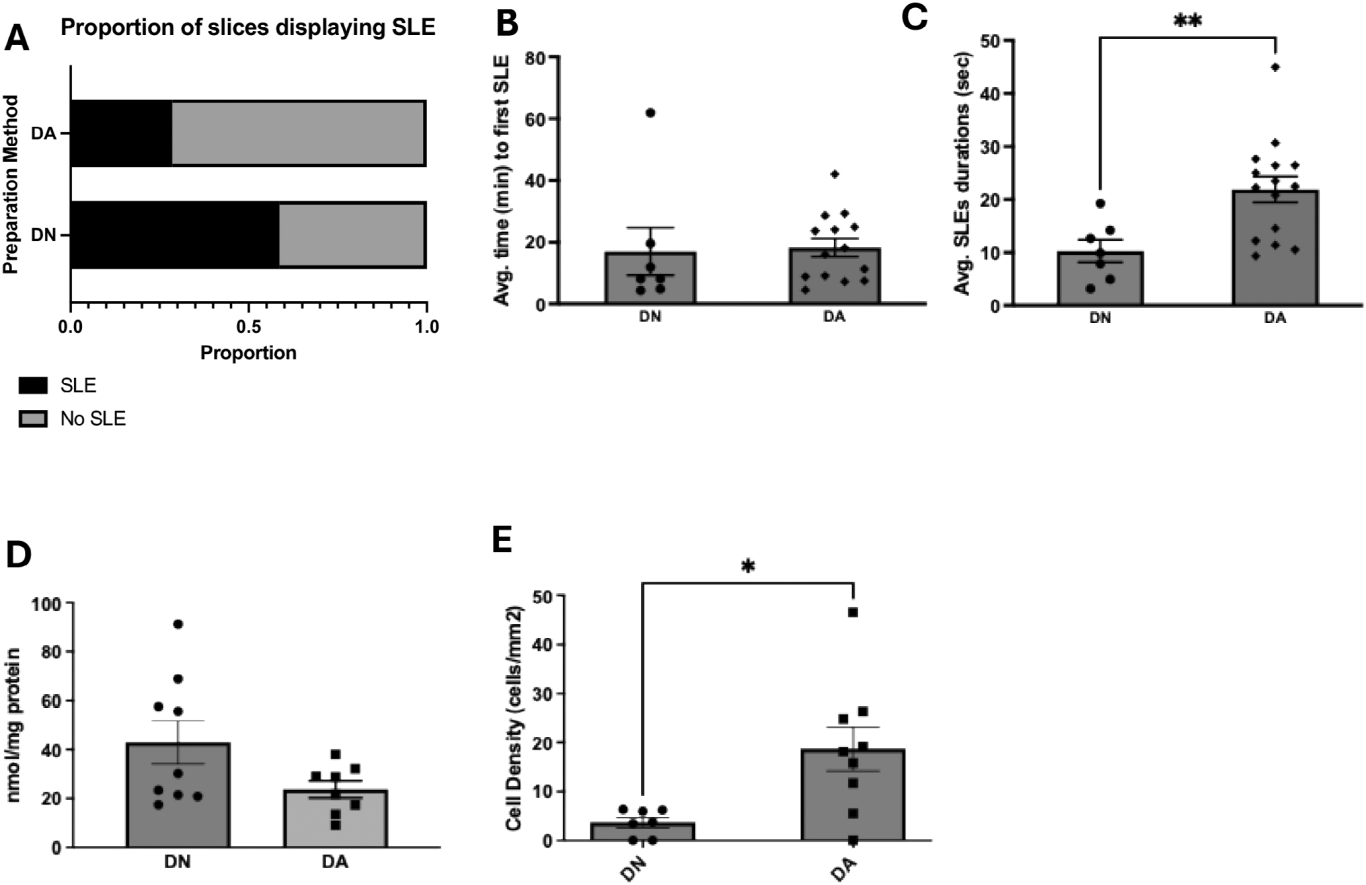
Seizure‐like event (SLE) induction is rescued by addition of antioxidants in the cutting solution. In panel A is shown the proportion of slices displaying SLE in mice prepared with decapitation in nACSF cutting solution supplemented with antioxidants (DA) compared against nACSF only cutting solution. Shown in B and C is the average time to first SLE (B) in minutes and average SLE durations (C) in seconds. Shown in D is the carbonyl concentration as a measure of protein peroxidation in brain slices. Shown in E is cell count of PV^+^ interneurons measured through immunostaining. DN, decapitated with nACSF; DA, decapitated with nACSF supplemented with antioxidants, * *p* < 0.05, **p < 0.01, ***p < 0.001, ****p < 0.0001, *n* for DN—7, DA—6. Chi‐square test was used for A, Mann–Whitney test was used for B, *t*‐test was used for C, D, and E. Number of slices (*n*) for DN = 7–9 slices from 7 rats, DA = 8–14 slices from 7 rats. Error bars represent SEM.

Addition of antioxidants in the DN cutting solution (see Figure 5) significantly affected the proportion of slices displaying SLEs, even if they are prepared through decapitation, *x* ^2^ (1, *N* = 21) = 7.8; *p* = 0.005. Specifically, the proportion of slices displaying SLEs in decapitated mice prepared in nACSF cutting solution containing antioxidants (28.6%) was significantly lower than mice prepared in nACSF cutting solution (58.6%). Interestingly, there is no significant difference (Mann Whitney, *U* = 35, *p* = 0.3223) in the average time to first SLE. However, there is a significant increase (unpaired two‐tailed *t*‐test, *t*(20) = 2.99, *p* = 0.0072) in the duration of SLEs from 12.2 ± 1.9 s to 21.8 ± 1.4 s, an SLE with duration more typical of the other slice preparation methods. We measured carbonyl concentrations as a measure of protein peroxidation (Levine et al. 1990), a readout of oxidative stress. As predicted, carbonyl concentration in the brain slices is reduced in the antioxidant‐treated brain slices, although this did not reach statistical significance (unpaired two‐tailed *t*‐test, *t*(15) = 1.94, *p* = 0.07). Finally, we examined the density of PV^+^ interneurons, which were significantly lost during the DN preparation. Interestingly, supplementation of antioxidants significantly rescued the interneuron loss (unpaired two‐tailed *t*‐test, *t*(14) = 2.88, *p* = 0.0122), increasing its cell density (DA, 18.7 ± 4.5 cells/mm^2^; DN, 3.7 ± 1.0 cells/mm^2^).

### 3.5 Oxidative Damage During Brain Slice Preparation Is Mediated by Hypoxic Stress Response

Antioxidant supplementation in the cutting solution partially mitigated the loss of inhibitory interneurons and the hyperexcitable network observed with the DN slice preparation method. This observation suggests that the loss of inhibitory interneurons can largely be attributed to oxidative damage during brain slice preparation. Given that oxidative stress is tightly linked with hypoxia (Merelli et al. 2021), we hypothesize that the hypoxic event during the decapitation procedure leads to this oxidative damage. To examine this hypothesis, we wanted to inhibit the hypoxic response pathway that regulates hypoxic stress response upstream of oxidative stress (Majmundar et al. 2010). This pathway is largely mediated by the hypoxia‐inducible factor protein (HIF‐1), with the HIF‐1α being responsible for the acute hypoxia response (Ziello et al. 2007). To modulate this pathway, we supplemented the cutting solution with chrysin, a known inhibitor of HIF‐1α (Fu et al. 2007).

**FIGURE 6:**
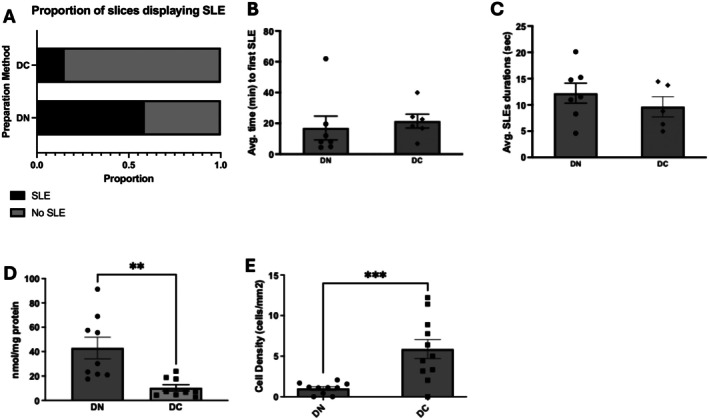
Seizure‐like event (SLE) induction is rescued by addition of chrysin, an inhibitor of HIF‐1α in the cutting solution. In panel A is shown the proportion of slices displaying SLE in mice prepared with decapitation in nACSF cutting solution supplemented with chrysin (DC) compared against nACSF only cutting solution. Shown in B and C is the average time to first SLE (B) in minutes and average SLE durations (C) in seconds. Shown in D is the carbonyl concentration as a measure of protein peroxidation in brain slices. Shown in E is cell count of PV^+^ interneurons measured through immunostaining. DN, decapitated with nACSF; DC, decapitated with normal aCSF supplemented with chrysin, * *p* < 0.05, **p < 0.01, ***p < 0.001, *n* for DN—7, DC—6. Chi‐squared test was used for A, Mann–Whitney test was used for B, C, and D, *t*‐test was used for E. Number of slices (*n*) for DN = 6–9 slices from 7 rats, DC = 6–10 slices from 4 rats. Error bars represent SEM.

Addition of chrysin to the cutting solution (see Figure 6) significantly affected the proportion of slices displaying SLEs, *x* ^2^ (1, *N* = 40) = 31.3; *p* < 0.0001. Specifically, the proportion of slices displaying SLEs in decapitated mice prepared in nACSF cutting solution containing chrysin (15%) was significantly lower than in mice prepared in normal aCSF cutting solution (58.6%). Interestingly, unlike the antioxidant cutting solution, we did not observe any significant difference in the dynamics of the SLE; neither the time to first SLE (Mann Whitney, *U* = 12, *p* = 0.2343) nor the average SLE durations (Mann Whitney, *U* = 12, *p* = 0.4318). Carbonyl concentration in brain slices prepared with cutting solution containing chrysin (DC; 10.4 ± 2.5 nmol/mg protein) was significantly lower (Mann–Whitney, *U* = 6, *p* = 0.0012) than in slices prepared with nACSF cutting solution (DN; 43.0 ± 8.8 nmol/mg protein). Finally, the density of PV^+^ interneurons was similarly significantly higher (unpaired two‐tailed *t*‐test, t(19) = 3.90, *p* = 0.0010) in slices prepared in cutting solution containing chrysin as compared against slices prepared in nACSF cutting solution (DC, 5.9 ± 1.2 cells/mm^2^; DN, 1.0 ± 0.2 cells/mm^2^).

## 4 Discussion

Ex vivo acute brain slice electrophysiology has been a mainstay in neuroscience research for the past 50 years and more (Li and McIlwain 1957) – yet, surprisingly, very few studies have comprehensively characterized the degree of neuronal cell preservation following acute brain slice preparation. We found that brain slice preparation method significantly affected the survival and preservation of neuronal population in the brain slice. In particular, we found that the combination of transcardial perfusion and the use of sACSF preserves the most viable neuronal population. Our current findings are consistent with previous reports showing that sACSF preserves neuronal populations more effectively than nACSF when used as a cutting solution (Aghajanian and Rasmussen 1989; Richerson and Messer 1995). However, interestingly, when brain slices were prepared using cervical dislocation followed by decapitation rather than transcardial perfusion, this difference becomes less apparent. This suggests that the method of using cervical dislocation followed by decapitation (a Schedule 1 method (UK Animals (Scientific Procedures) Act 1986)) induces a baseline level of damage to brain slices, which could not be mitigated using sACSF. In the framework of the 3Rs (Lee et al. 2020), transcardial perfusion with sACSF can be considered a refinement, as it serves as a protective technique that enhances the preservation of brain circuitry, particularly GABAergic interneurons. This experimental refinement is important for critical in vitro brain slice physiological studies where neuronal network functions (Kuenzi et al. 2000) are to be examined. This refinement can be specifically attributed to the use of the brain slice preparation method as other parameters that can influence neuronal viability such as temperature, slicing angle, or vibratome parameters were kept constant in this current study.

The density of inhibitory and excitatory neuronal populations was diminished during brain slice preparation; however, the use of transcardial perfusion in combination with sACSF significantly mitigated this neuronal loss. We predicted that inhibitory interneurons, in particular PV+ interneurons, could be rapidly lost to oxidative damage during brain slice preparation from its inherent high metabolic nature (Wang and Michaelis 2010); but we had not anticipated that excitatory neurons would be similarly lost during brain slice preparation. However, cervical dislocation causes rapid respiratory arrest (Carbone et al. 2012), and the subsequent hypoxia‐ischemia is known to bring about damage and cell death in cortical pyramidal neurons (Akulinin et al. 1997; Barakat et al. 2024; Sadowski et al. 1999). Markedly increased extracellular glutamate levels during hypoxia (López‐Pérez et al. 2012), combined with impaired glutamate reuptake (Vicente et al. 2009), are also likely to contribute to excitotoxic conditions that underlie the significant neurodegeneration of glutamatergic neurons observed. The degeneration of layer III pyramidal neurons in the entorhinal cortex was especially pronounced. This finding may be due to the high levels of recurrent excitatory synaptic connections between these cells and a relatively high degree of electrical coupling (Dhillon and Jones 2000). Early loss of inhibitory interneurons in neurodegeneration has also been shown to lead to a later loss of excitatory neurons in the brain (Maestú et al. 2021; Montañana‐Rosell et al. 2024; Tweedy et al. 2021), suggesting a bilateral relationship between inhibitory loss and excitatory neurodegeneration. It is interesting to note that in both human and pre‐clinical animal models of temporal lobe epilepsy, neurodegeneration in layer III of the mEC is a key feature (Du et al. 1995; Du and Schwarcz 1992; Du et al. 1993; Kim et al. 1990).

Immunocytochemical analysis of specific inhibitory interneuron subtypes revealed that different brain slice preparation methods preferentially preserved distinct interneuron populations. Using the most effective neuronal preservation method—transcardial perfusion with sACSF—PV^+^ and CB^+^ interneurons showed the highest levels of preservation. This finding was unsurprising given these cell types' high metabolic activity (Gulyás et al. 2006) and the vulnerability of PV^+^ interneurons to elevated glutamatergic tone and associated oxidative stress (Behrens et al. 2007; Cantu et al. 2015). NMDAR expression on PV^+^ interneurons also contributes to the selective vulnerability of this cell type in this context. PV^+^ interneurons in the superficial layers of the mEC have a significant synaptic NMDA component (Jones and Bühl 1993) in comparison to other PV^+^ interneurons in other cortical regions. Excessive glutamate is known to activate NMDARs on PV^+^ interneurons and cause an abnormal influx of calcium ions. This high intracellular calcium burden will in turn lead to neuronal damage/death via a combination of oxidative stress, energy failure and activation of proteolytic enzymes. CB^+^ interneurons have more heterogeneous expression of mitochondrial respiratory enzymes. However, in temporal lobe regions such as the mEC, their mitochondrial expression is as high as parvalbumin‐expressing interneurons (Gulyás et al. 2006). Interestingly, decapitation with sACSF specifically preserved VIP^+^ and CCK^+^ interneurons. It is unclear why these specific subclasses of interneurons are preserved by decapitation with sACSF; but CCK^+^ and VIP^+^ interneurons are known to have their own circuitry (Nguyen et al. 2020).

Given the significant differences in neuronal populations across various brain slice preparation methods, we next examined the neurophysiological dynamics in ex vivo brain slice electrophysiological recordings. Consistently, brain slices prepared with nACSF, particularly when combined with cervical dislocation and decapitation, exhibited characteristics of hyperexcitability. The probability of successfully inducing seizure‐like events (SLEs) using an acute epilepsy model was significantly higher in brain slices prepared via cervical dislocation and decapitation. In the case of the 0 Mg^2+^ model, a progressive breakdown of GABAergic inhibition plays a crucial role in the evolution of induced epileptic activity (Whittington et al. 1995). This previous finding suggests that for this particular acute seizure model to work a reduction in levels of GABAergic inhibition is required. In the present study, this alteration in inhibition is reflected by the decreased proportion of PS slices generating SLEs and the significant time taken for SLEs to occur in contrast to DN slices. Both these parameters are shown to be linked to the degree of inhibition in the brain as feedforward inhibition has been shown consistently to modulate seizure dynamics (Liou et al. 2018; Trevelyan and Schevon 2013). Thus, the preservation of inhibitory interneurons in the ‘more viable’ brain slice preparation method has not only affected the success of SLE induction in in vitro acute epilepsy model; but also inherently changed the SLEs induced in these brain slices. Thus, yet again, another philosophical question arises whether SLEs induced in conditions of ‘high inhibition’ are similar in nature to SLEs induced in conditions of ‘low inhibition’ in the brain slice.

To identify the mechanism of neuronal loss during brain slice preparation, we devised several rescue experiments. Given the high metabolic rate of parvalbumin‐expressing interneurons (Gulyás et al. 2006) and their susceptibility to oxidative damage (Wang and Michaelis 2010), we hypothesized that the loss of neurons during brain slice preparation is primarily due to oxidative stress. Indeed, application of a cocktail of antioxidants in the nACSF cutting solution significantly attenuated the loss of parvalbumin‐expressing interneurons—increasing the parvalbumin interneuron population by six‐fold, which subsequently corrected the hyperexcitability traits of these brain slices. Importantly, the SLE dynamics is also more akin to the profile of sACSF‐prepared slices; indicating a potent preservation of inhibition by antioxidant addition to nACSF. Given the period of anoxia experienced by the brain in the decapitation procedure, we suspected that the hypoxia response pathway may be upstream of the oxidative stress experienced by the brain slices (Majmundar et al. 2010). To test this, we applied HIF‐1α inhibitors in the nACSF cutting solution and while this also rescued parvalbumin‐expressing interneurons and the hyperexcitability in the slice, it does not significantly change the profile of the SLEs induced. While we are not certain why the dynamics of SLEs are unchanged by the addition of a HIF‐1α inhibitor, HIF‐1α is known to modulate many other biological processes; including autophagy and apoptosis (Chen et al. 2022); in addition to its central role in oxidative stress response. Collectively, this indicates that oxidative stress is central to the loss of neurons incurred during brain slice preparation and this may be partially regulated by HIF‐1α‐dependent mechanisms. Likely, there are other factors at play that collectively contribute to the damage incurred during brain slice preparation such as HIF‐2‐dependent mechanism (Ratcliffe 2007), excitotoxicity (Schurr et al. 1995), and mitochondrial dysfunction (Fried et al. 2014).

Regardless, our study has raised an important question with wide‐ranging implications for neuroscience research. The method by which brain slice preparation is conducted significantly affects the neuronal population preserved in the brain slice and ultimately, the network physiology and dynamics. Thus far, many brain slice studies conducted have not considered the impact of their brain slice preparation method on the interpretations of their results. This is especially important when considering the popular use of ex vivo brain slice preparation in acute epilepsy model (Morris et al. 2023), pharmacological studies (Burman et al. 2019), and metabolic studies (Qi et al. 2021). Given our results that indicate a higher likelihood of generating epileptiform activity with decapitation model, it is unsurprising that many studies, even to this day, still utilize brain slices prepared with decapitation with nACSF in acute ex vivo epilepsy studies (Cerovic et al. 2023; Dong et al. 2020; Gonzalez‐Sulser et al. 2011). While the use of this brain slice preparation method offers a reliable success in the induction of SLEs, our study raised an important question—whether these SLEs are ‘artificially’ induced by a particularly damaging brain slice preparation method that does not preserve inhibition. This is especially interesting as some of these brain slice models are lauded as in vitro drug‐resistant epilepsy model (Burman et al. 2019; Cerovic et al. 2023) but given the lack of preservation of physiological inhibition in these brain slices, the lack of pharmacological response of some of these anticonvulsant drugs would need to be re‐evaluated with this consideration in mind.

While this study comprehensively evaluated the metabolic changes in brain slice preparation, there are factors that we had not examined in the current study. Male rats were exclusively used in this study due to the known effect of estrogen on brain slice electrophysiology and epileptic threshold in the brain (Ledoux et al. 2009; Li et al. 2024). Future studies should expand on the present findings to examine if oxidative stress damage during brain slice preparation is modulated in the presence of an active estrous cycle in female rodents. Furthermore, this study was conducted in the context of modeling adult epilepsy by focusing on rats aged 10–12 weeks old. Rodent brain slices obtained from juvenile or aged animals are known to have distinct electrophysiological properties and thus, their use in the context of the brain slice preparation described here needs to be examined further. A recent study also found that microglia and inflammation are significantly affected by different brain slice preparation methods (Berki et al. 2024). Given the significant metabolic change with different brain slice preparation methods, we suspected that glial cells would be significantly affected as well in our models, and this could be an important future direction for our study. Another crucial factor in brain slice preparation is the temperature of the cutting solution. In our current study, we maintained this variable at ice‐cold temperature, a popular choice in brain slice preparation; but some studies have shown that using cutting solutions at physiological temperatures also significantly changed the neuronal dynamics in the brain slice (Eguchi et al. 2020; Huang and Uusisaari 2013); arguably closer to a physiological state. Temperature significantly alters metabolic state and provides another variable to examine in future studies.

## 5 Conclusion

Our study is, to our knowledge, the first to comprehensively characterize the impact of different brain slice preparation methods on the preservation of neuronal population and their network dynamics in the resultant brain slices. We found that the combination of transcardial perfusion of rodents and the use of sucrose‐based artificial cerebrospinal fluid as cutting solution is the best brain slice preparation method to preserve inhibition in the brain slice. Arguably, this preservation of inhibition is a crucial goal for brain slice preparation to model physiological brain states. The loss of inhibitory interneurons incurred in brain slice preparation that leads to this loss of inhibition is due to oxidative stress experienced during brain slice preparation. Limiting this oxidative stress during brain slice preparation can preserve inhibition in the brain and should be considered a neuroprotective strategy during ex vivo brain slice preparation. This has a broad impact for the use of ex vivo brain slice preparation for epilepsy studies and future studies using this popular model should consider the impact that the brain slice preparation has on the physiological and metabolic state of the tissue.

## Author Contributions

**Felix Chan:** conceptualization, methodology, formal analysis, data curation, investigation, writing – original draft, writing – review and editing, supervision, visualization. **Anupam Hazra:** methodology, formal analysis, investigation, data curation, supervision. **Ashan Jayasekera:** investigation, data curation. **Katherine Huang:** data curation, investigation. **Shuna Whyte:** investigation, data curation. **Leolie Telford‐Cooke:** data curation, investigation. **Kamilah Lakhani:** investigation, data curation. **Xiaomeng Li:** investigation, data curation. **Rebecca Shields:** data curation, investigation. **Angeline Kosim:** investigation, data curation. **Darwin Su:** data curation, investigation. **Carol Murray:** methodology, investigation, data curation, supervision. **Mark O. Cunningham:** conceptualization, methodology, formal analysis, investigation, resources, data curation, writing – original draft, writing – review and editing, visualization, supervision, project administration, funding acquisition.

## Funding

This work was supported by Wellcome Trust, 102037. Science Foundation Ireland, 16/RC/3948, 20/FFP‐P/8613. Engineering and Physical Sciences Research Council, A000026, EP/K50499X/1.

## Conflicts of Interest

Dr. Felix Chan is a handling editor at Journal of Neurochemistry and a member of International Society for Neurochemistry. All other authors declare no conflicts of interest.

## Supporting information


**Table S1:** List of primary antibodies used in this study.
**Table S2:** List of secondary antibodies used in this study.

## Data Availability

All datasets generated and analyzed during this study are available from the corresponding principal investigators upon reasonable request.
