# Supplementary material for: Inhibitory GABAergic Neuron Loss due to Oxidative Damage During Ex Vivo Acute Brain Slice Preparation Influences Genesis and Dynamics of Epileptiform Activity
Source: J Neurochem. 2026 Jan 28;170(2):e70367. doi: 10.1111/jnc.70367 (PMC12848643; doi:10.1111/jnc.70367)
Supplement: Supplementary file 1 — Table S1: List of primary antibodies used in this study. Table S2: List of secondary antibodies used in this study. [file JNC-170-0-s001.pdf]

Inhibitory GABAergic neuron loss due to oxidative damage during *ex vivo* acute brain slice preparation influences genesis and dynamics of epileptiform activity

Felix Chan, Anupam Hazra, Ashan Jayasekera, Katherine Huang, Shuna Whyte, Leolie Telford-Cooke, Kamilah Lakhani, Xiaomeng Li, Rebecca Shields, Angeline Kosim, Darwin Su, Carol Murray, and Mark O. Cunningham

Supplementary Table 1. List of primary antibodies used in this study

Target protein	RRID	Supplier	Product code	Host species	Dilution
NeuN	AB_2341095	Merck Millipore	ABN90P	Guinea pig	1:1000
GABA	AB_476667	Sigma Aldrich	A0310	Mouse	1:1000
CaMKII	AB_868641	Abcam	AB52476	Rabbit	1:1000
GFAP	AB_2109645	Merck Millipore	AB5804	Rabbit	1:1000
PV	AB_477329	Sigma Aldrich	P3088	Mouse	1:1000
CB	AB_10000340	Swant	CB38	Rabbit	1:10,000
CR	AB_10000342	Swant	CG1	Goat	1:1000
Somatostatin	AB_2302603	Santa Cruz	SC7819	Goat	1:1000
VIP	AB_2216428	Merck Millipore	AB982	Rabbit	1:1000
CCK	AB_2228728	Proteintech	13074-2-AP	Rabbit	1:500

Supplementary Table 2. List of secondary antibodies used in this study

Antibody	RRID	Supplier	Product code
Biotinylated goat-anti rabbit IgG antibody	AB_2313606	Vector Laboratories	BA-1000
Biotinylated horse-anti mouse IgG antibody	AB_2313581	Vector Laboratories	BA-2000
Biotinylated horse-anti goat IgG antibody	AB_2336123	Vector Laboratories	BA-9500
Biotinylated goat-anti guinea pig IgG antibody	AB_2336132	Vector Laboratories	BA-7000
